# Country‐Specific Participation Patterns in Transnational Governance Initiatives on Sustainability: Preliminary Insights and Research Agenda

**DOI:** 10.1002/gch2.202300012

**Published:** 2023-07-06

**Authors:** Jale Tosun, Emiliano Levario Saad, Johannes Glückler, Alejandra Irigoyen Rios, Rosa Lehmann

**Affiliations:** ^1^ Institute of Political Science Bergheimer Straße 58 69115 Heidelberg Germany; ^2^ Institute of Geography Berliner Straße 48 69120 Heidelberg Germany; ^3^ Heidelberg Center for Ibero‐American Studies Brunnengasse 1 69117 Heidelberg Germany

**Keywords:** climate change, environment, global governance, Latin America, sustainability, transnational public–private governance initiatives

## Abstract

Transnational public–private governance initiatives (TGIs) have become key elements in global governance, especially in the governance of sustainability. Pertinent research has concentrated on why TGIs have emerged as well as on their impacts on political outcomes and questions related to their legitimacy. This instructive literature has predominantly focused on TGIs as entities in their own right. This explorative study contributes to the literature by advocating a complementary analytical perspective that pays attention to domestic‐level patterns of participation in TGIs and national factors that determine which types of organizations (public, business, or civil society) participate in TGIs. It is shown for six Latin American countries (Argentina, Brazil, Chile, Colombia, Mexico, and Peru) that there exists cross‐country variation in the composition patterns in 29 TGIs on sustainability, suggesting that national conditions matter for how organizations participate in them. By improving the knowledge of the national conditions, a more complete analysis of participation and the effectiveness of TGIs can be provided in global sustainability governance. In this spirit, in a last step, an agenda is developed for guiding future research on this topic.

## Graphical abstract

This study assesses how public, business, and civil society organizations based in Argentina, Brazil, Chile, Colombia, Mexico, and Peru participate in transnational governance initiatives (TGIs) on sustainability. The participation patterns vary across the six Latin American countries, suggesting that domestic causes and consequences of engagement at the transnational level warrant enhanced attention by future research.

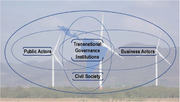

## 1 Introduction

The United Nations Conference on Environment and Development held in 1992 established the notion of sustainable development as an attempt to align economic, environmental, and social concerns in ways that retain today's opportunities for future generations.^[^ ^1^, ^2^ ^]^ This conference also created different types of governance arrangements, one of which is transnational public–private governance initiatives (TGIs). These are institutions in which public actors and/or intergovernmental organizations cooperate with private actors, most importantly, business and civil society, with the purpose of governing transnational problems,^[^ ^3^ ^]^ thus pooling governance authority across the public and private spheres.^[^ ^4^ ^]^ The emergence of TGIs in the 1990s has been compared to a “Cambrian explosion.”^[^ ^5^ ^]^ Regardless of whether one agrees with this characterization, we are witnessing the existence of a governance landscape, which some have denoted as “fragmented,”^[^ ^6^, ^7^ ^]^ others as “complex,”^[^ ^8^, ^9^ ^]^ and yet others as “polycentric,”^[^ ^10^, ^11^ ^]^ in which TGIs are important elements for governing environmental issues and climate change as well as sustainability more generally.^[^ ^12^ ^]^

In this study, we build on the extensive literature on TGIs^[^ ^3^, ^5^, ^13^, ^14^ ^]^ and related concepts such as “transnational multi‐stakeholder partnerships,”^[^ ^15^, ^16^, ^17^, ^18^, ^19^, ^20^ ^]^ “transnational public–private partnerships,”^[^ ^4^ ^]^ “(complex) global governance,”^[^ ^6^, ^8^, ^21^, ^22^, ^23^, ^24^ ^]^ and “(transnational) hybrid governance,”^[^ ^25^, ^26^, ^27^ ^]^ to name just a few. We advocate an analytical perspective that concentrates on the different types of bodies—that is, public, business, and civil society organizations—which make up TGIs.

We consider the country‐focused approach rewarding for two reasons. First, in order to better understand how TGIs perform their governance functions at the transnational level, it is useful to assess what kind of organizations partake in them.^[^ ^5^ ^]^ The composition of TGIs depends on the decision of individual organizations to take part in them. From this perspective, TGIs can be regarded as an opportunity structure, which organizations can choose to use in order to achieve their organizational goals. The decision by organizations to become engaged in TGIs depends on domestic conditions.^[^ ^4^, ^28^, ^29^, ^30^ ^]^ Evidently, organizational decision‐making includes other factors as well, such as the features of a given TGI,^[^ ^6^, ^31^ ^]^ and international factors such as the influence of organizations providing development aid, to give an example for developing countries.^[^ ^14^, ^28^ ^]^ However, in the literature, domestic factors have received less attention vis‐à‐vis international factors;^[^ ^12^ ^]^ to advance the state of research, it therefore appears reasonable to concentrate on this set of factors.

As stated above, TGIs can be deemed as providing opportunity structures for organizations willing to engage in transnational sustainability governance. They are, by design and definition, accessible to public, business, and civil society organizations. It is by facilitating the exchange among different types of organizations that TGIs can have an impact on the governance of sustainability issues.^[^ ^3^ ^]^ We know for the transnational level that different types of organizations partake in them.^[^ ^15^ ^]^ However, our knowledge is limited concerning the participation of different types of organizations based in the same countries. In other words, do all organization types make use of the opportunity structure offered by a given TGI on sustainability? If TGIs on sustainability brought together a diverse set of organizations, this could have positive impacts for the domestic governance of sustainability issues, too. As far as we are aware, country clusters of organizations engaging in TGIs on sustainability have received limited attention—a research gap which this study identifies and aims to reduce.

What are the country‐specific patterns for how public, business, and civil society organizations partake in TGIs on sustainability? What are the domestic factors that account for the potential country‐specific patterns? To answer our research questions, we analyze an original dataset which captures how public, business, and civil society organizations based in Argentina, Brazil, Chile, Colombia, Mexico, and Peru partake in TGIs on sustainability. Our conception of public organizations includes those based at the central (e.g., federal ministries), state (e.g., regional governments), and local (e.g., cities and municipalities) levels. We define business actors as for‐profit organizations governed by private law, whereas civil society organizations are nonprofit organizations governed by private law. We chose these countries because of cross‐country variation regarding, first, the policy competences of public organizations at the central, state, and local levels; second, the degree to which they are economically globalized; third, the level of foreign direct investment; and fourth, the influence of civil society organizations. At the same time, all six countries are presidential systems, which facilitates their comparison by holding constant this important institutional variable that structures politics and governance.^[^ ^32^, ^33^, ^34^, ^35^, ^36^ ^]^

The remainder of this study unfolds as follows. First, we outline in greater detail what TGIs are and what functions they fulfill in global sustainability governance. Then, we review the literature and summarize which domestic conditions have been found to impact participation in TGIs on sustainability. Subsequently, we present our methodological approach, and then follow the presentation and discussion of our empirical findings, which feed into a section in which we detail an agenda for future research. In the final section, we summarize our main insights and offer some concluding reflections.

## 2 Transnational Public–Private Governance Initiatives

In International Relations, the dominant notion of cooperation, until the end of the Cold War, focused on states as the main actors. This “methodological nationalism”^[^ ^37^, ^38^ ^]^ was superseded by the notion of global governance, which, similar to other governance concepts, acknowledges the role played by public as well as private actors and international and transnational organizations.^[^ ^23^ ^]^ It is against the background of the emergence of a dedicated literature on global governance that TGIs have increasingly attracted scholarly interest.

TGIs are organized initiatives in which public and private actors agree to collectively commit resources to jointly pursue objectives of transnational concern and impact. Given their transnational nature, environmental issues and climate change represent prime examples of areas governed by TGIs.^[^ ^12^ ^]^ This also holds true for sustainability more generally, which includes, in addition to the environmental dimension, economic and social concerns.^[^ ^22^, ^26^ ^]^

In general, following Westerwinter,^[^ ^3^ ^]^ TGIs share four characteristics: first, a TGI must involve at least one public and/or intergovernmental organization, one business organization (firms, business associations, and foundations), and one civil society actor (nongovernmental organizations (NGOs), NGO coalitions, and universities or other research institutes); second, the activities of TGIs must be of a transnational nature; third, a TGI must perform tasks that concern the governing of a transnational problem; fourth, a TGI needs to be institutionalized to the degree that it can facilitate regular interactions between its participants.

TGIs are attributed different functions for governing issues transnationally, including agenda setting, information sharing, capacity building, the adoption and application of soft and hard forms of regulation, and integration across different governance arenas.^[^ ^39^ ^]^ Other sources add monitoring, funding and service provision, and knowledge creation and transfer to the governance functions of TGIs.^[^ ^3^ ^]^

Agenda setting has been discussed widely in the pertinent literatures, including policy studies and comparative politics. In essence, agenda setting refers to processes by which issues are included in the public, media, and/or political agenda.^[^ ^40^ ^]^ The pertinent literature argues that of these three agenda types, the political one is the most consequential because it means that an issue is up for serious consideration by policymakers and thus can end up being addressed by a public policy. To give an example, the Brazilian State of São Paulo used its membership in TGIs to bring issues regarding climate change and biodiversity onto the agenda of the Conference of the Parties of the corresponding framework conventions of the United Nations.^[^ ^41^ ^]^

Information sharing and knowledge creation relate to the fact that TGIs provide venues for communication among different types of stakeholders and stakeholders located at different levels of political systems.^[^ ^42^ ^]^ From this perspective, since TGIs bring together participants from different governance arenas (e.g., different policy sectors or different levels of government), they can facilitate the development of a more holistic understanding of a given issue and help produce solutions that are equally more holistic and create co‐benefits. Information sharing and knowledge creation within TGIs can stimulate or facilitate lesson drawing and policy learning.^[^ ^43^ ^]^ The exchange of resources and the provision of funding can result in capacity building.^[^ ^44^ ^]^ Research has shown, for example, that engagement in TGIs has increased the capacity and influence of women's nongovernmental organizations in Latin America.^[^ ^45^ ^]^

Compared to the above functions, soft or hard regulation of the participants can entail that they commit themselves to adopting and implementing measures, which can be tailor‐made to the individual participant or represent a jointly formulated measure regarding given sustainability issues.^[^ ^46^ ^]^ Monitoring and service provision can be directed toward the TGI participants or external audiences, such as states and how well they comply with the pledges they made to the 2015 Paris Agreement on Climate Change^[^ ^47^ ^]^ or how they support the implementation of transnational forest projects.^[^ ^7^, ^48^, ^49^ ^]^ For example, transnational city networks on climate change, such as the Covenant of Mayors for Climate & Energy, have instruments in place for policy tracking and surveillance, which facilitate the implementation of ambitious local‐level climate policies and measures.^[^ ^46^ ^]^

In addition to these direct functions, TGIs aiming to influence the behavior of the participants toward explicit sustainability goals can also fulfill indirect roles through discursive and normalizing practices, which entail the exchange of ideas and beliefs.^[^ ^39^ ^]^ Another function attributed to TGIs is to increase the legitimacy of international politics since they facilitate the representation of different stakeholders.^[^ ^50^ ^]^ For example, Bernstein and Cashore argue that international norms and discourse played a vital role in enabling the different stakeholders in forestry to agree on the concept of sustainable forest management.^[^ ^8^ ^]^

Overall, the above overview demonstrates that TGIs can perform important governance functions. In terms of their effectiveness for problem‐solving, the findings in the literature are mixed. Individual TGIs, such as the Global Alliance for Vaccines and Immunization and the Forest Stewardship Council, have been shown to be effective,^[^ ^20^ ^]^ whereas other partnerships have a limited track record.^[^ ^15^, ^51^ ^]^ One of the conditions for effective governance, identified by Pattberg and Widerberg,^[^ ^15^ ^]^ is the social and political context in which TGIs are situated.

In our view, it is necessary to elaborate on this point and develop a more comprehensive understanding of country‐specific participation patterns, as this will allow us to understand governance dynamics both at the transnational and national levels better and explain the performance of TGIs in terms of their ability to solve a given sustainability‐related problem or problem complex.

## 3 Domestic Drivers of Engagement in TGIs on Sustainability

The literature has already put forth the argument that domestic conditions matter for participation in TGIs on sustainability. For example, Andonova focuses on the participation of countries in such initiatives and contends that domestic factors such as capacity, together with the constituencies of transnational actors, international donors, and institutions, are important for explaining whether countries partake in them.^[^ ^4^ ^]^ Andonova and Sun reveal that domestic concerns such as climate vulnerability and advancing renewable energy, in addition to institutions such as the Clean Development Mechanism and targeted foreign aid, shape the states’ engagement in transnational voluntary carbon offset programs.^[^ ^28^ ^]^ Concentrating on firms’ decisions on whether to participate in TGIs, Hsueh stresses the importance of domestic regulatory contexts, which condition the business actors’ strategic considerations.^[^ ^29^ ^]^

When shifting focus to civil society organizations and the domestic conditions for their participation in TGIs, research has stressed the possibilities of “venue shopping”^[^ ^52^, ^53^ ^]^ and “forum shopping”^[^ ^24^ ^]^ offered by the multilevel^[^ ^54^, ^55^, ^56^ ^]^ or polycentric^[^ ^10^, ^11^, ^57^, ^58^ ^]^ nature of the governance landscape. According to the concepts of multilevel and polycentric governance (for a discussion, see ref. [58 ^]^), there exist multiple levels where decisions are taken, which offer an opportunity for actors to select from a set of institutional venues, including transnational ones, in the hope of finding receptive audiences in order to advance their own interests.^[^ ^4^, ^53^, ^59^, ^60^ ^]^ Research has portrayed civil society organizations as inclined to participate in transnational governance arrangements because their influence is limited at the national level—this corresponds to the perspective put forth by Pralle,^[^ ^53^, ^61^ ^]^ for instance.

Based on the concept of venue or forum shopping, we expect the ability of civil society organizations to influence public policy in a given country to affect how well they are represented in TGIs on sustainability (Expectation 1).

Similar arguments have been advanced with regard to subnational and substate actors who support more ambitious sustainability‐related policies but cannot influence decision‐making at the national level.^[^ ^62^, ^63^, ^64^, ^65^ ^]^ Hence, they engage in TGIs on sustainability in order to “bypass” national governments and realize their policy goals, as well as to exert pressure on national governments to adopt stricter policies via the transnational level.^[^ ^61^, ^66^ ^]^

Drawing again on the notion of venue or forum shopping, we expect competences of subnational/state‐level and substate/local‐level organizations in a given country to affect how well they are represented in TGIs on sustainability (Expectation 2).

As concerns business organizations, partaking in TGIs on sustainability can be motivated by several factors. Sustainability could be seen as a challenge that is best mastered by getting involved in TGIs. Conversely, sustainability could be perceived by firms as an opportunity to develop and implement new technologies and business models.^[^ ^67^ ^]^ The literature has shown that large firms are more likely to become engaged in TGIs than small ones because their operations produce more environmental emissions^[^ ^67^ ^]^ as well as because they can afford to participate in them in terms of their financial and organizational capacity. Among the large firms, multinational enterprises (MNEs), that is, firms that sustain and operate subsidiaries in more than one country are considered to be the ones that are most likely to participate in TGIs, since civil society organizations tend to focus their monitoring and mobilization actions on them.^[^ ^68^ ^]^ MNEs are also targeted by domestic regulatory standards and public policies more generally.

Given the increased societal and regulatory attention directed toward MNEs,^[^ ^22^, ^69^ ^]^ we expect this type of business to be most the likely to join a TGI on sustainability (Expectation 3). However, since regulatory standards and public contestation vary across countries, we anticipate the existence of cross‐country variation in the participation patterns of MNEs.

## 4 Materials and Methods

To identify the TGIs to be included, we drew on the Transnational Public–Private Governance Initiatives in World Politics dataset compiled by Westerwinter,^[^ ^3^ ^]^ which contains information on 636 TGIs. From this population, we included those TGIs that focus on sustainability issues and in which at least one actor from Argentina, Brazil, Chile, Colombia, Mexico, and Peru participates. This coding approach aligns with our conception of TGIs as opportunity structures open to public, business, and civil society organizations, should they choose to take advantage of them. From this, it follows that all TGIs included in this analysis have a membership at the transnational level that brings together public, business, and civil society actors. However, when looking at the organizations based in a given country, it is possible that not all three organization types are represented. In our view, this is an observation worth reporting since it may, as we explain later, have implications for how TGIs can influence national governance for sustainability.

Based on the data published by Westerwinter, we identified 26 TGIs as working on sustainability. We added three TGIs (Renewable Energy and Energy Efficiency Partnership, Renewable Energy Policy Network for the 21st Century, and Low Emission Development Strategies Global Partnership) identified by Weischer et al.,^[^ ^70^, ^71^ ^]^ giving us 29 in total. We know of other datasets that pay more attention to subnational and substate actors, such as the dataset by Bulkeley et al. on transnational initiatives for governing climate change.^[^ ^39^ ^]^ But given our interest in assessing country‐specific patterns, the TGIs identified by Westerwinter^[^ ^3^ ^]^ and Weisch et al.^[^ ^70^ ^]^ provide an appropriate empirical basis. When taking forward with this line of inquiry, it would nonetheless be useful to merge the various datasets.

**Table** **1** gives an overview of the TGIs included. They cover a wide range of sustainability issues. Climate change and renewable energy feature prominently as TGI work topics and are addressed by the Climate Group, the Global Alliance for Climate‐Smart Agriculture, the LEDS Global Partnership, the Greenhouse Gas Protocol, the Joint Implementation Network Climate and Sustainability, the Global Methane Initiative, the Cities Climate Finance Leadership Alliance, 4/1000 Initiative, Connect4Climate, Renewable Energy and Energy Efficiency Partnership, Renewable Energy Policy Network for the 21st Century, and the Low Emission Development Strategies Global Partnership.

**Table 1:** TGIs included in the analysis (*N* = 29)

No.	Name
1	Climate Group
2	United Nations Environment Program (UNEP) Finance Initiative
3	Carbon Pricing Leadership Coalition
4	Global Alliance for Climate‐Smart Agriculture
5	Low Emission Development Strategies (LEDS) Global Partnership
6	The New York Declaration on Forests
7	Tropical Forest Alliance
8	Sustainable Development Solutions Network
9	World Resources Institute
10	International Emissions Trading Association
11	WIPO Green
12	Sustainable Coffee Program
13	Greenhouse Gas Protocol
14	Forest Investment Program
15	International Association of Public Transport
16	Global Agenda for Sustainable Livestock
17	International Maize and Wheat Improvement Centre
18	Joint Implementation Network Climate and Sustainability
19	Global Methane Initiative
20	Tourism 2030
21	Global Forum on Agricultural Research
22	Cities Climate Finance Leadership Alliance
23	4/1000 Initiative: Soils for Food Security and Climate
24	Committee on Sustainability Assessment
25	Global Alliance for Buildings and Construction
26	Connect4Climate
27	Renewable Energy and Energy Efficiency Partnership^a)^
28	Renewable Energy Policy Network for the 21st Century^a)^
29	Low Emission Development Strategies Global Partnership^a)^

^a)^ TGIs identified on the basis of Weischer et al.^[^ ^70^ ^]^

Other topics addressed by the TGIs are buildings and construction, agriculture, forestry, tourism, and investment. In addition, some TGIs concentrate on sustainability defined broadly, such as the Sustainable Development Solutions Network or the World Resource Institute. The Sustainable Development Solutions Network is one of the TGIs that is particularly visible among both academics and practitioners alike and is considered to present an epistemic community in the sense of Haas.^[^ ^55^, ^72^ ^]^ It was founded in 2012 to deliver integrated approaches for implementing the Sustainable Development Goals and the Paris Agreement on Climate Change.

In a second step, we visited the TGIs’ websites to gather information on their participants. Following the approach of Westerwinter, we differentiated between public, business, and civil society organizations.^[^ ^3^ ^]^ For the six states selected, this coding decision produced a dataset of 706 observations.

As stated in the previous section, the analysis rests on three expectations regarding the influence of domestic conditions. **Table** **2** provides key statistics on the six Latin American states, including their population and the level of affluence as measured by the Gross Domestic Product (GDP) per capita. In addition, the table reports the scores of the Regional Authority Index (RAI), which is a granular operationalization of whether countries are federal or unitary. The RAI captures the degree to which authority is exercised by a subnational government over those who live in the region, as well as the degree to which the authority is exercised by a subnational government or its representatives in the country as a whole^[^ ^73^ ^]^—information we require to evaluate Expectation 1. The higher the score, the more authority the subnational governments have, which will be high in federal states and low in unitary ones. How effectively civil society can participate politically is captured by the Civil Society Index (CSI) by the V‐Dem project,^[^ ^74^ ^]^ which is an indicator that allows us to assess Expectation 2. Higher values indicate a higher level of civil society participation in a given country. Turning to Expectation 3, net inflows of Foreign Direct Investment (FDI) and the degree of economic globalization, as reported by the Swiss Economic Institute (KOF) globalization index,^[^ ^75^ ^]^ indicate the expected prevalence of MNEs in a given country. Higher values suggest that a country is more economically globalized and receives FDI, which should entail a stronger presence of MNEs.

**Table 2:** Key statistics

Country	Population, 2023 [million]^a)^	GDP per capita, 2021 [US$]^b)^	CSI, 2021 (0–100)^c)^	RAI (2018) [0–30]	FDI, net inflows, 2021 [% of GDP]^d)^	KOF globalization score, 2020 [0‐100]^e)^
Argentina	45	10 636	87	24.50	1.4	46
Brazil	212	7507	75	21.79	2.9	42
Chile	19	16 265	86	6.00	4.0	67
Colombia	51	6104	64	15.01	3.0	48
Mexico	128	10 045	44	21.14	2.6	60
Peru	22	6.62	75	22.06	3.3	58

^a)^ https://www.worldometers.info/world‐population/population‐by‐country/

^b)^ https://data.worldbank.org/indicator/NY.GDP.PCAP.CD

^c)^ https://v‐dem.net/data/the‐v‐dem‐dataset/country‐year‐v‐dem‐core/

^d)^ https://data.worldbank.org/indicator/BX.KLT.DINV.WD.GD.ZS

^e)^ https://kof.ethz.ch/en/forecasts‐and‐indicators/indicators/kof‐globalisation‐index.html.

In the third step, we assigned each of these three organization types to more refined categories, which we chose in line with the pertinent literature. To classify the public organizations, we created three categories according to the literature on multilevel sustainability governance:^[^ ^54^ ^]^

- *Central bodies*: Government, ministries, governmental bodies, and national branches of international organizations
- *State bodies*: Government, ministries, and governmental bodies
- *Local bodies*: Cities and municipalities

While we will not test a dedicated expectation, we still consider it instructive to provide more granular information on the types of civil society organizations to bolster our argument that there are country‐specific participation patterns in TGIs. To classify civil society organizations, we adopted a simplified version of the International Classification of Non‐profit Organizations (ICNPO) created by Salamon and Anheier.^[^ ^76^, ^77^ ^]^ The classification focuses on sustainability‐focused civil society organizations and their main activity areas, which we assigned to five categories:

- Education and research
- Environment
- National branches of international NGOs
- Business/professional associations and unions
- Others

The “other” category comprises a wide range of organizations such as those working on culture, development, and social affairs, which we observed very few times and therefore decided not to treat as separate categories.

For business organizations, the operationalization is particularly straightforward since the dataset simply differentiates between MNEs and national companies. The firms were assigned to either of the categories based on the information they provided on their websites.

The dataset comprises binary variables assigned to the different coding categories, which are used to produce count variables or metric variables capturing percentage shares. The data were produced by two coders who completed a total of three coding rounds. We analyze the data descriptively by means of graphs and summary statistics.

## 5 Empirical Analysis

The empirical analysis consists of two steps. First, we inspect the overall country‐specific participation patterns in TGIs. In a second step, we offer more nuanced insights into the participation patterns observed.

**Figure 1:**
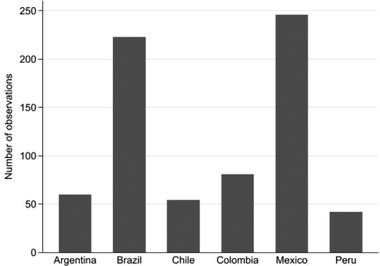
Participation in TGIs by country, total number (minimum 54 to maximum 247).

**Figure** **1** shows the total number of organizations (public, business, and civil society) based in the countries which participate in the 29 TGIs on sustainability (see Tables S1–S6 in the Supporting Information). We can infer from the figure that the participation of organizations is highest for those based in Mexico and lowest for those based in Peru. This pattern is plausible and roughly aligns with the population size of the countries (see Table 2), with the exception that Brazil has a higher population than Mexico but ranks second.

**Figure 2:**
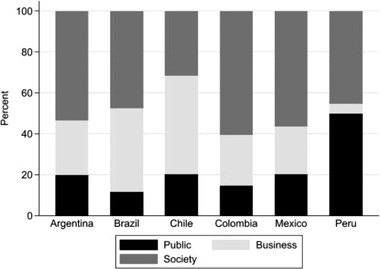
Participation in TGIs by organization type and country, in percentage shares.

The empirical picture is even more interesting when inspecting the country‐specific participation patterns as shown by **Figure** **2**. In Chile, business organizations dominate among the TGI participants, followed by civil society. The smallest group represented among Chilean members is the public sector. The composition of Peruvian participants is exceptional as it has a very high share of public organizations and a very low share of business organizations. The patterns for Argentine, Brazilian, Colombian, and Mexican TGI participants resemble each other regarding the dominance of civil society organizations over the other organization types.

**Figure 3:**
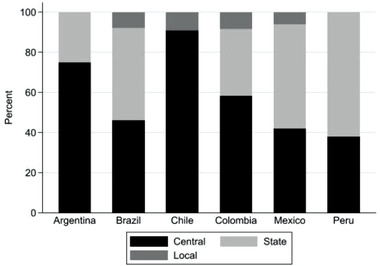
Participation in TGIs by public organizations, in percentage shares.

**Figure** **3** provides a more refined picture of the public organizations and their participation in TGIs on sustainability. The data reveal that organizations based at the central level dominate the participation patterns of Argentina, Chile, and Colombia. The most extreme case is Chile, where almost 91% of the public organizations participating in TGIs are located at the central level, followed by Argentina with 75% of the public bodies located at the central level, and Colombia with 58%. Furthermore, among the Chilean participants there is not a single organization based at the state level. It is also worth noting that Argentine and Peruvian cities and municipalities do not participate in the TGIs on sustainability selected for this analysis. The Peruvian pattern is also interesting because central‐level organizations are outnumbered by those located at the state level, which also holds true for Brazilian and Mexican TGI participants.

**Figure 4:**
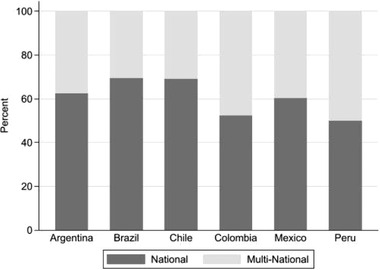
Participation in TGIs by business organizations, in percentage shares.

**Figure** **4** provides insights into the business organizations participating in the TGIs on sustainability, distinguishing between national enterprises and MNEs. The data presented here are intriguing for two reasons. First, we can see that, with the exception of Peru, the share of national businesses partaking in these TGIs is higher than it is for MNEs. As concerns Peru, there are only two business organizations that are members of TGIs on sustainability; one of these is a national company and the other is an MNE. Thus, the participation pattern of Peru is less interesting in respect of the equal share of national firms and MNEs participating in the TGIs, but it is intriguing that very few firms based in the country are engaged at the transnational level. Second, although we do see differences in the participation patterns for the countries, these are statistically insignificant (see Table S7 in the Supporting Information). Thus, the cross‐country variation observed could be due to chance.

**Figure 5:**
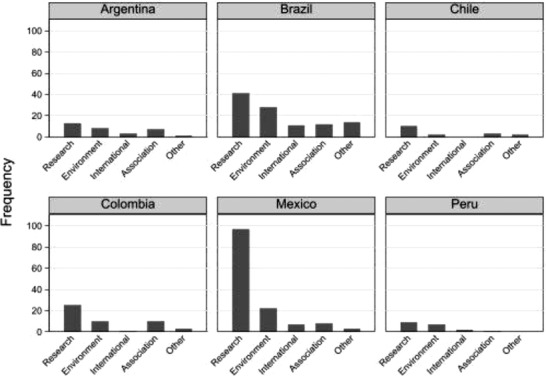
Participation in TGIs by civil society organizations.

Next, we turn to the participation patterns of civil society organizations. **Figure** **5** provides two interesting insights. The first is that the TGI participation patterns share a similarity across all countries, as education and research institutions dominate. As Tables S1–S6 in the Supporting Information reveal, several universities are members of TGIs for sustainability, which is reflected in the participation patterns. Second, in Brazil, Mexico, and Peru, the second‐most represented group is environmental organizations, whereas in Argentina, Chile, and Colombia, this group is composed of professional associations and unions.

Overall, the nuanced assessment of civil society organizations suggests that there are signs of both cross‐country similarities and differences between the TGI participants from different countries. The empirical observations suggest that factors accounting for cross‐country variation appear as worthy of examination as those accounting for similarities by means of appropriate theoretical frameworks and methods.

## 6 Discussion

According to Expectation 1, the ability of civil society organizations to influence public policy in a given country should determine how well they are represented in TGIs on sustainability. These abilities are shaped by the specific features of a political system which provides political opportunity structures.^[^ ^78^ ^]^ In some countries, the context is favorable for civil society actions and thus they can attempt to influence national policymakers directly, for example, by participating in parliamentary hearings. In other countries, the context is less favorable, and civil society organizations must shift to other forums in order to make an impact, including engagement in TGIs.^[^ ^24^, ^53^, ^59^ ^]^

As shown by Table 2, the CSI scores indicate that Argentina and Chile offer the most favorable national contexts for civil society organizations, and Mexico the least favorable one. Contrasting this information with the share of civil society organizations in Figure 2, we see that there is no marked difference between the participation patterns in TGIs on sustainability for Argentina and Mexico. However, there is a stark difference between civil society organizations based in Chile and Mexico and how they are represented in the TGIs selected. Following the logic of venue or forum shopping, the observations for Chile and Mexico are plausible. Given their CSI, Chilean civil society organizations can be perceived to be better integrated in the country's political processes and therefore do not need to bypass domestic policymaking by engaging in TGIs. By contrast, alone the institutional characteristics of the country's political system seemingly make it more likely for Mexican civil society organizations to find themselves in a position where they need to shop transnationally for venues or forums. However, the fact that civil society organizations based in Argentina, a country with favorable conditions according to its CSI score, also engage transnationally suggests that the reasoning underlying this expectation requires refinement.

Instead of concentrating on the opportunity structure of civil society organizations for influencing domestic policy, it appears reasonable to contrast their sustainability policy preferences with the sustainability policies actually adopted by the governments, as this will better enable us to determine their motivation for going transnational.^[^ ^53^, ^61^ ^]^ Likewise, the capacity of civil society organizations is a factor that can be hypothesized to affect their decisions to engage in TGIs on sustainability^[^ ^79^ ^]^—a factor we have not taken into consideration here.

Expectation 2 postulated that the varying competences of subnational/state‐level and substate/local‐level organizations should result in cross‐country variation in how these participate in TGIs on sustainability. As highlighted above, this expectation was confirmed (see Figure 3). To explain this variation, two aspects seem important: first, the degree to which actors at this political level have policymaking competences; second, the policy preferences of state bodies vis‐à‐vis those of central bodies.^[^ ^34^, ^56^ ^]^ The RAI scores presented in Table 2 illustrate that Chile is the country in which the policy‐making competences of the state bodies are most constrained (it has the lowest RAI score). At the same time, we have observed that no Chilean state body participates in the TGIs selected. This is reasonable because public bodies at this level of the political system do not have the power to enact policies. The Chilean regions do play a role in defining sustainability policies but mostly in the frame of the regional development strategies,^[^ ^63^ ^]^ which the policymakers at the subnational level must negotiate with the central‐level government. This specific relationship between bodies at the central and state levels could explain the pattern observed for Chile.

Figure 3 suggests that state bodies in Peru are particularly well represented in TGIs. Although Peru does not have the highest RAI score of the six countries, it is the country in which state bodies have become empowered via decentralization.^[^ ^62^ ^]^ In situations in which subnational actors become endowed with political power they tend to be keen to use it, as one could observe for the climate policy adopted by the Scottish government after devolution, for example.^[^ ^64^ ^]^ The Peruvian case could be similar, and it could well be the case that during the process of decentralization subnational policymakers contested policy decisions made by the central government.^[^ ^62^ ^]^ Extant research shows that Peruvian state bodies prefer more ambitious sustainability policies than the central bodies, but the latter is more powerful. Therefore, it is plausible that Peruvian state bodies participate in TGIs in order to access a forum via which they can collaborate with external actors and use them to increase pressure on the central government.^[^ ^4^ ^]^

Expectation 3 postulated that MNEs are more likely to participate in TGIs on sustainability than national firms, and that because of the different levels of FDI and globalization there should be cross‐country differences regarding this aspect, too. While we could observe cross‐country differences, these were not statistically significant. The expected differences between the two types of firms equally did not turn out to be as marked as expected. Even more importantly, in all countries except Peru, the share of national firms participating in TGIs on sustainability was higher than the share of MNEs (see Figure 4). While this finding deviates from our expectation, it needs to be explored in more empirical detail, which would have gone beyond the scope of this analysis. We differentiated between national firms and MNEs; however, another indicator would have been to identify small‐ and medium‐sized enterprises (SMEs) among the national firms. 99.5% of the economies in Latin American are considered to be SMEs.^[^ ^80^ ^]^ While national companies dominate the participation patterns for businesses, one needs to consider that even if their number is higher than that of MNEs, so is the population of SMEs in the region, too. Another expectation that requires further attention refers to the economic sectors of the companies, as these can have important implications for their decision to engage in TGIs on sustainability.^[^ ^29^, ^69^ ^]^

Overall, our overarching argument about the existence of cross‐country variation in the TGI participation was confirmed. This was the main point we sought to convey. As concerns the explanations for the cross‐country variation observed, the reasoning we presented in Section 3 requires further refinement.

## 7 Future Research Perspective: Feedbacks on Domestic Politics and Governance

The empirical analysis has concentrated on the question of whether there exists cross‐country variation in how the different organizations participate in TGIs and has proposed some domestic‐level variables for explaining them. However, our analysis also offers insights that could help to explain whether and to what extent engagement in TGIs on sustainability has implications for domestic sustainability governance, as this would resonate with the basic tenet of multilevel governance.^[^ ^29^, ^54^, ^55^, ^56^, ^58^ ^]^

As **Table** **3** shows, there is variation across countries and TGIs as to whether only one, two, or all three types of organizations participate in them. The variation observed aligns with our reasoning that TGIs provide opportunity structures which are accessible to all three types of organizations, but that the decision of whether to engage is ultimately up to the organization.

**Table 3:** Overlaps between the different actor types from the same country in TGI participation (P: public organization; B: business organization; and C: civil society organization; TGIs' names are shortened but can be inferred from Table 1)

No.	Name	Argentina	Brazil	Chile	Colombia	Mexico	Peru
1	The Climate Group	P	P/B	P	P	P/B	P
2	UNEP Finance Initiative	B	B	B	B	B	
3	Carbon Pricing Leadership…		B	B	P/B/C	P/B	
4	Global Alliance for Climate…		B/C	P	C	P/B/C	
5	LEDS Global Partnership	C		P/B/C		B/C	
6	New York Declaration on…	P	P	P	P/C	P/B/C	P/C
7	Tropical Forest Alliance		B/C		P/C		P
8	Sustainable Development…	B/C	P/B/C	C	C	P/C	B/C
9	World Resources Institute		C			C	
10	International Emissions…		B				
11	WIPO Green		P	P		B	
12	Sustainable Coffee Program		P/B/C		B/C	C	B
13	Greenhouse Gas Protocol					B	
14	Forest Investment Program		C			B	C
15	International Association…	B	P/B	B	B	P/B	
16	Global Agenda for…	P/B/C	C		C	B/C	
17	International Maize…	C	B/C	C	C	P/B/C	P/C
18	Joint Implementation…			B/C		B	
19	Global Methane Initiative	P/B/C	P/B/C	P/B	P/B/C	P/B/C	P
20	Tourism 2030	P/C	P/B/C	B/C	P/B/C	P/B/C	
21	Global Forum on…	C	P/B/C		B/C	C	C
22	Cities Climate Finance…	P	P				
23	4/1000 Initiative: Soils for…	P/B/C		P/B/C		P/B/C	
24	Committee on Sustainability…				C	P	C
25	Global Alliance for Buildings…	P/C	P/B	P/B/C	P/B	P/C	P
26	Connect4Climate		B	C	B	P/C	
27	Renewable Energy and…	P/B		P		P/B/C	
28	Renewable Energy Policy…	B	P			P	
29	Low Emission…	C		P/B/C		P/B/C	

A constellation in which all three organization types participate in TGIs can be analytically interesting for several theoretical reasons. One of them, and arguably the most interesting one, is that in such constellations, conflicting viewpoints on sustainability issues among domestic actors could potentially be overcome because TGIs facilitate communication and conflict resolution between actors.^[^ ^57^, ^81^ ^]^ For example, civil society organizations could use TGIs to ask policymakers to increase the level of ambition of sustainability policies, while making sure that business actors support them in this demand.

As we can infer from Table 3, the members of the Global Methane Initiative based in Argentina, Brazil, Colombia, and Mexico comprise all three types of organizations. As concerns Chilean participation, public and business organizations are represented, and in the case of Peru only public organizations. We suggest that addressing methane emissions could be more effective in those countries where all three organization types are represented, because their joint membership in the Global Methane Initiative could stimulate mutual learning and result in a convergence of their policy preferences regarding this issue.

Another research perspective worth pursing that is related to the domestic effects of TGI engagement would be to assess the behavior of those organizations in TGIs which are not joined by other organization types, that is, which represent only public, business, or civil society actors from one country. This is the case for the participation of Argentine civil society organizations in LEDS Global Partnership, the International Maize and Wheat Improvement Centre, the Global Forum on Agricultural Research, and the Low Emission Development Strategies Global Partnership. One could assess whether engagement in TGIs has empowered these organizations vis‐à‐vis other organizations in the domestic policy process or in domestic sustainability governance. Or one could hypothesize that the domestic implications of TGIs are more limited if only one organization type participates in them.

Likewise, constellations in which business and civil society actors are represented in TGIs appear interesting since these could result in a situation in which their policy preferences converge, and policymakers therefore engage in anticipatory policymaking and proactively propose stricter sustainability standards. Empirically, such a constellation is given for the participation of Colombian organizations in the Sustainable Coffee Program or for the Peruvian organizations that engage in the Sustainable Development Solutions Network.

## 8 Conclusion

Societies are constantly confronted with challenges of a global scale, particularly when these concern climate change and environmental protection, energy, food supply, water provision and safety, health and healthcare, technological development, and sustainability. The latter has been on the international political agenda since the mid‐1980s. In addition to actions taken by national governments and international processes led by international organizations such as the United Nations, TGIs play an important role for achieving sustainability.^[^ ^3^, ^4^, ^5^, ^12^, ^14^, ^17^, ^28^, ^30^, ^39^, ^44^, ^47^ ^]^ They are based on governance arrangements that entail collaboration and a re‐articulation of roles across public and private actors,^[^ ^4^ ^]^ which appear necessary for several characteristics of the challenges concerned such as “telecoupled” systems.^[^ ^82^ ^]^ Given their number and varying characteristics, scholars from different disciplines have investigated TGIs with regard to their features and composition^[^ ^3^, ^5^, ^13^, ^66^ ^]^ and their effectiveness.^[^ ^15^, ^16^, ^20^ ^]^

Acknowledging the insights provided by this literature, with this study we advocate a research perspective that takes the multilevel nature of the global governance landscape seriously and pays attention to country‐specific participation patterns in TGIs on sustainability. We contend that by embracing such a perspective, we can better understand how domestic factors determine the organizations’ engagements with them as well as gauge which consequences TGIs have for policymaking and governance at the domestic level. In line with our overall expectation, we could show that there exists intriguing cross‐country variation regarding the engagement of public, business, and civil society organizations based in Argentina, Brazil, Chile, Colombia, Mexico, and Peru in the 29 TGIs on sustainability investigated.

Despite the insights provided, the analysis provided here is of an indicative nature only and characterized by limitations. The most important limitation is the small number of TGIs and countries investigated, which would need to be expanded when taking this research agenda forward. Another limitation concerns the lack of nuance in the coding of business organizations, which we consider to be the reason why no statistically significant cross‐country variation could be observed. Lastly, the concepts discussed for explaining cross‐country variation need to be revisited, and expanded, as do the mechanisms explaining how TGIs can have an impact on domestic policymaking and governance.

Overall, we are aware that this study can only mark a starting point for research that investigates the domestic reasons for why organizations join TGIs on sustainability and what feedback their engagement produces at the domestic level. We hope that this study will serve as the point of departure for future research that ventures in this direction.

## Conflict of Interest

The authors declare no conflict of interest.

## Supporting information

Supporting Information

## Data Availability

The data that support the findings of this study are openly available in heiDATA at 10.11588/data/Q8HKD2, reference number "Country‐Specific Participation Patterns in Transnational Governance Initiatives on Sustainability".
