# Supplementary material for: Country‐Specific Participation Patterns in Transnational Governance Initiatives on Sustainability: Preliminary Insights and Research Agenda
Source: Glob Chall. 2023 Jul 6;7(8):2300012. doi: 10.1002/gch2.202300012 (PMC10448143; doi:10.1002/gch2.202300012)
Supplement: Supplementary file 1 — Supporting Information [file GCH2-7-2300012-s001.pdf]

Global Challenges
Open Access

Supporting Information

for *Global Challenges.*, DOI 10.1002/gch2.202300012

Country-Specific Participation Patterns in Transnational Governance Initiatives on Sustainability: Preliminary Insights and Research Agenda

Jale Tosun, Emiliano Levario Saad, Johannes Glückler, Alejandra Irigoyen Rios and Rosa Lehmann*

Table S1: Argentina-based TGI members

**Public Organizations**<br>(Entities governed by public law, which are located at the central, state, and local level, and national representations of international organizations)<br>• Santa Fe<br>• Tucumán<br>• Provincia de Misiones / Misiones Province<br>• Instituto Nacional de Tecnología Agropecuaria / National Institute of Agricultural Technology<br>• Gobierno de Argentina / Government of Argentina<br>• Centro de Zoologia Aplicada Argentina / Argentine Applied Zoology Center<br>• Red Argentina de Municipios frente al Cambio Climático / Argentine Network of Municipalities against Climate Change<br>• Instituto Nacional de Tecnología Agropecuaria Argentina / National Institute of Agricultural Technology Argentina<br>• Ministerio de Medio Ambiente y Desarrrollo Sostenible / Ministry of Environment and Sustainable Development
**Business Organizations**<br>(Entities governed by private law and which engage in for-profit activities)<br>• Banco de Galicia y Buenos Aires SA / Bank of Galicia and Buenos Aires S.A.<br>• Banco de la Nación Argentina (BNA) / Bank of the Argentine Nation<br>• Banco Bilbao Vizcaya Argentaria (BBVA) / Bank Bilbao Vizcaya Argentaria<br>• Emova Movilidad S.A. / Emova Mobility S.A.<br>• Cámara de Comercio Italiana en la República Argentina / Italian Chamber of Commerce in the Argentine Republic<br>• Enerco2<br>• Quality Tonnes<br>• UNO Ingeniería / UNO Engineering<br>• Vallmitjana & Company Consulting Services<br>• Logiseed<br>• PUMA - Plataforma Inteligente de Manejo Agricola S.A. / Intelligent Platform for Agricultural Management S.A.<br>• Ruuts S.A<br>• Magellan Australia<br>• Fundación Bariloche / Bariloche Foundation<br>• Unión de Industriales para Saneamiento Cuencas Matanza Riachuelo y Reconquista (UISCUMARR) - Union of Industrialists for Sanitation of Matanza Riachuelo and Reconquista Basins
**Civil Society Organizations**<br>(Entities governed by private law and which engage in non-profit activities as defined by Salamon and Anheier 1996)<br>• Asociación Sustentar / Sustentar Association<br>• Instituto de Investigaciones en Energía no Convencional (INENCO) / Non-Conventional Energy Research Institute<br>• Instituto para la Participación y el Desarrollo (INPADE) / Institute for the Participation and the Development<br>• Facultad Regional Córdoba de la Universidad Tecnológica Nacional / Córdoba Regional Faculty of the National Technological University

- Nuestras Huellas
- Asociación de Amigos de la Patagonia **/** Patagonian Friends Association
- Ingenieria sin Fronteras Argetina / Engineering Without Borders Argentina
- Universidad Nacional de Raúl Scalabrini Ortiz / National University of Raúl Scalabrini Ortiz
- Universidad de San Andrés, Facultad de Educación, Programa de Educación para la Sustentabilidad / University of San Andres, School of Education, Education for Sustainability Program
- Centro de Desarrollo Sustentable GEO, FCE – UBA / Center for Sustainable Development
- Instituto Interdisciplinario de Economía Política de Buenos Aires / Interdisciplinary Institute of Political Economy of Buenos Aires
- Universidad Nacional de la Patagonia Austral / National University of Southern Patagonia
- Redes Chaco / Networks Chaco
- Consejo Nacional de Investigaciones Científicas y Técnicas / National Council for Scientific and Technical Research
- Universidad de Buenos Aires / Univeristy of Buenos Aires
- Asociacion para el Estudio de los Residuos Sólidos (ARS) / Argentina Solid Waste Association
- Fundacion Torcuato Di Tella / Foundation Torcuato Di Tella
- Universidad del Norte Santo Tomás de Aquino / Saint Thomas Aquinas University Of The North
- Movimiento Agrario Misionero/ Pámpano Desarrollo Turístico / Agrarian Missionary Movement/ Pámpano Tourism Development
- Fundación Plan 21 / Plan 21 Foundation
- Fundacion Ecoturismo Argentina / Argentine Ecotourism Foundation
- Universidad de Congreso / University of Congreso
- Comunidad Huerta SAS / Orchard Community
- Asociación Argentina de Productores en Siembra Directa (AAPRESID) / Argentine Association of Direct Seed Producers
- Regeneration International
- Centro Argentino de Inginieros (CAI) / Argentine Center of Engineers
- Asociación Sustentar / Sustentar Association

Table S2: Brazil-based TGI members

**Public Organizations**<br>(Entities governed by public law, which are located at the central, state, and local level, and national representations of international organizations)<br>• Acre<br>• Amapá<br>• Amazonas<br>• Mato Grosso<br>• Pernambuco<br>• Rondônia<br>• São Paulo City<br>• São Paulo State<br>• Tocantins<br>• Acre, Brazil<br>• Amapa, Brazil<br>• Amazonas, Brazil<br>• Gerência de Mudanças Climáticas e Desenvolvimento Sustentável da Secretaria Municipal de Meio Ambiente da Cidade do Rio de Janeiro / Management of Climatic Changes and Sustainable Development of the Municipal Secretary of the Environment of the City of Rio de Janeiro<br>• National Institute of Industrial Property (INPI Brazil)<br>• SEAPA – Secretaria de Agricultura, Pecuária e Abastecimento de Minas Gerais / Ministry of Agriculture, Livestock and Supply of Minas Gerais<br>• Companhia Do Metropolitano De Sao Paulo – Metro / Sao Paulo Metropolitan Company - Metro<br>• Governo do Brasil / Government of Brazil<br>• Ministério do Meio Ambiente / Environment Ministry, Brazil<br>• Rio Grande Do Sul Rice Institute / Rice Institute of Rio Grande do Sul<br>• BDMG<br>• Cidade de São Paulo / City of Sao Paulo<br>• Centro de Operações Rio / Rio Operations Center<br>• Instituto Pereira Passos / Institute Pereira Passos<br>• UN-Habitat - Regional Office for Latin America and the Caribbean
**Business Organizations**<br>(Entities governed by private law and which engage in for-profit activities)<br>• Unidas<br>• Banco Bradesco S.A.<br>• Banco BTG Pactual SA<br>• Banco de Amazônia SA<br>• Banco Itaú Holding Financeira S.A.<br>• Banco Nacional de Desenvolvimento Economico e Social (BNDES) / National Bank for Economic and Social Development<br>• Bradesco Seguros / Bradesco Insurance<br>• Brasilcap Capitalização S.A<br>• Brasilseg<br>• Caixa Econômica Federal<br>• Caixa Seguradora SA<br>• Itau Seguros S.A / Itau Insurance S.A.

- Liberty Seguros S/A / Liberty Insurance S.A.
- MONGERAL AEGON Seguros e Previdencia S.A.
- Porto Seguro S.A.
- Seguradora Lider DPVAT
- Sompo Seguros S.A / Sompo Insurance S.A
- SulAmérica
- CPFL Energia
- Pecsa
- NatCap
- Marfrig
- JBS
- ABN AMRO
- Empresa de Obras Publicas (EMOP) / Public Works Company
- Unicarbo
- Associação Brasileira da Industria de Café (ABIC) / Brazilian Coffee Industry Association
- Associação Brasileira da Indústria de Café Solúvel (ABICS) / Brazilian Soluble Coffee Industry Association
- Associação dos Produtores de cafés Especiais do Circuit (ACECAP) / Association of Circuit Specialty Coffee Producers
- Associação dos Cafeicultores de Montanha de Divinolândia (APROD) / Association of Mountain Coffee Growers of Divinolândia
- Atlântica Exportação e Importação Ltda / Atlântica Exportation and importation
- Cecafé – Conselho dos Exportadores de Café do Brasil / Council of Coffee Exporters from Brazil
- Conselho Nacional do Café (CNC) / National Coffee Council
- Cooperativa de Cafeicultores e Agropecuaristas (Cocapec) / Cooperative of Coffee Growers and Farmers
- Comexim Ltda.
- Companhia Têxtil de Castanhal / Textile Company of Chestnut
- Cooperativa Agrária dos Cafeicultores de São Gabriel (Cooabriel) / Agrarian Cooperative of Coffee Growers of São Gabriel
- Cooperativa de Produção dos Cafeicultores do Cerrado de Araguari (Coocacer) Ltda. / Cerrado de Araguari Coffee Growers Production Cooperative
- Cooperativa Mista Agropecuária de Paraguaçu Ltda. (Coomap) / Mixed Agricultural Cooperative of Paraguaçu
- Cooperativa Agropecuária Centro Serrana (Coopeavi) / Centro Serrana Agricultural Cooperative
- Coopervitae
- Exportadora de Café Guaxupé / Guaxupé Coffee Exporter
- FalCafé Comércio Exportação Importação de Café Ltda. / FalCafé Trade Export Import of Coffee
- Itapuan Coffees
- Minasul – Cooperativa dos Cafeicultores da Zona de Varginha / Minasul –Varginha Zone Coffee Growers Cooperative
- Pinhalense S/A Máquinas Agrícolas / Pinhalense S/A Agricultural machinery
- Tristão Cia. de Comércio Exterior Ltda. / Tristão Co. of Foreign Trade
- ALSTOM Brazil
- Bombardier Transportation Brazil

- Bombardier Transportation Sao Paulo
- Indra Brasil Soluções E Serviços Tecnologicos Ltda / Indra Brasil Technological Solutions and Services
- MAN Latin America
- Mercedes-Benz Do Brasil Ltda
- SIEMENS Mobility Soluções De Mobilidade / SIEMENS Mobility Solutions
- Systra Engenharia E Consultoria Ltda / Sytra Engineering and Consulting
- VOLVO do Brasil Veiculos Ltd / VOLVO from Brazil Vehicles
- ZF DO BRASIL LTDA
- AgE Tecnologias Meio Ambiente, Saneam. & Ambiência / AgE Technologies Environment, Sanitation. & Ambience
- Carbotrader
- DDMA Engenharia, Pesquisa e Desenvolvimento Ltda / Engineering, Research and Development Ltd
- Environmental Resources Management, Inc.
- GB Eco Solutions
- Green Domus Desenvolvimento Sustentável Ltda / Green Domus Sustainable development
- Your Way Ecotourism
- Cristalino Jungle Lodge
- Projeto bagagem
- Pousada candomba
- Travessia
- Maris
- Araras Eco lodge
- Recanto Ecológico Rio da Prata
- Estância Mimosa Ecoturismo
- International Tourism Development and Marketing Consulting
- Tocorime
- Trip on Jeep Ecoturismo
- Cormorano
- Gondwana Brasil
- Brazil Ecojourneys
- Pedra Afiada
- BirdView
- Horizon 2020 Consortium for Innovation (H2C-I)
- Brazilian Sustainable Construction Council
- Grupo ACA Alberto Couto Alves, S.A.
- Ex change for change Brasil
- Sao Paolo Film Commission
- ABIQUIM - Brazilian Chemical Industry Association
- Brazilian Tree Industry (IBA)
- Brazilian Rural Society
- Natura
- Brazilian Agricultural Research Corporation (Embrapa)

Civil Society Organizations
(Entities governed by private law and which engage in non-profit activities as defined by Salamon and Anheier 1996)

- Brazilian Association of Airlines (ABEAR)
- Conselho Empresarial Brasileiro para o Desenvolvimento Sustentável CEBDS / Brazilian Business Council for Sustainable Development
- Amigos da Terra – Amazônia Brasileira / Friends of the Earth – Brazilian Amazon
- Amazon Environmental Research Institute (IPAM)
- Imaflora
- ICV
- Dialog Instituto / Dialog Institute
- Instituto Ethos / Ethos Institute
- Enactus Brasil
- Institution for Transportation and Development Policy (ITDP)
- Universidade da Integração Internacional da Lusofonia Afro-Brasileira / University of International Integration of the Afro-Brazilian Lusophony (UNILAB)
- Conservation International (CI-Brazil)
- EMBARQ Brasil
- Sebrae-RJ - Serviço de Apoio às Micro e Pequenas Empresas no Estado do Rio de Janeiro / Support Service for Micro and Small Companies in the State of Rio de Janeiro
- Universidade Federal do Rio Grande do Norte / Federal University of Rio Grande do Norte
- Redes da Maré – Associação Redes de Desenvolvimento da Maré / Redes da Maré - Maré Development Networks Association
- Pontifical Catholic University of São Paulo (PUCSP)
- Programa Social Crescer e Viver / Crescer e Viver Social Program
- Instituto Nacional de Pesquisas da Amazonia (INPA) / National Research Institute of the Amazon
- Grupo de Institutos, Fundações e Empresas (GIFE) / Group of Institutes, Foundations and Companies
- CEBDS Brazil - Brazilian Business Council on Sustainable Development
- Instituto Internacional para Sustentabilidade / International Institute for Sustainability
- Universidade Rural Federal do Rio de Janeiro (URFRJ) / Federal Rural University of Rio de Janeiro
- Desafio Baanko
- Sindicato dos Trabalhadores Rurais na Agricultura Familiar de São Joaquim e Região (SINTRAF-SJR) / Union of Rural Workers in Family Agriculture of São Joaquim and Region
- Universidade Federal do Rio de Janeiro (UFRJ) / Federal University of Rio de Janeiro
- Observatório de Favelas do Rio De Janeiro / Observatory of Favelas in Rio De Janeiro
- Global Urban Development (GUD)
- Mamiraua Institute for Sustainable Development (MISD)
- Instituto Um Pé de Biblioteca /A Foot in the Library Institution
- CasaE-UFRGS (Universidade Federal do Rio Grande do Sul) / Federal University of Rio Grande do Sul
- Instituto Akatu / Akatu Institute
- Fundação Vitória Amazônica / Vitória Amazônica Foundation
- Wylinka

- Rede Global de Aprendizagem para o Desenvolvimento / Global Learning for Development Network
- Cities Alliance (Latin America and Caribbean office)
- Brazilian Cyclists' Union
- Centro de Estudos em Sustentabilidade (GVces) da Escola de Administração de Empresas da Fundação Getulio Vargas (FGV-EAESP) / Center for Sustainability Studies (GVces) of the Business Administration School of the Getulio Vargas Foundation (FGV-EAESP)
- Pontifícia Universidade Católica do Rio de Janeiro (PUC-Rio) / Pontifical Catholic University of Rio de Janeiro (PUC-Rio)
- Sistema B
- Fundo Brasileiro de Biodiversidade (FUNBIO) / Brazilian Biodiversity Fund
- Rede de Ação Política pela Sustentabilidade / Political Action Network for Sustainability
- Peabiru Institute
- Centro Universitário Newton Paiva / Newton Paiva University Center
- Comitê das Agendas 21 / Agenda 21 Committee
- Instituto de Estudos do Trabalho e Sociedade / Institute of Work and Society Studies
- Casa Fluminense
- Amazon Regional Network
- Bolsa Verde do Rio de Janeiro – BVRio / Environmental Exchange of Rio de Janeiro
- Associação Brasileira de Arquitetos Paisagistas (ABAP) / Brazilian Association of Landscape Architects
- Fundação Brasileira para o Desenvolvimento Sustentável / Brazilian Foundation for Sustainable Development
- Energy Efficiency and Sustainability Research Centre (Greens)
- Amazonas Sustainable Foundation (FAS)
- Roberto Marinho Foundation
- Instituto-E
- Comitê para a Democratização da Informática (CDI) / Committee for the Democratization of Information Technology
- Partnership Platform for the Amazon (PPA)
- Federal University of Amazonas
- Puxirum Institute of Sustainability and Energy Efficiency
- Universidade Nilton Lins – Manaus / Nilton Lins University - Manaus
- University Center Federal District
- Federal University of Paraiba (Centre for Public Policies and Sustainable Development)
- Perene Institute
- Formato Sustentável (formerly ODS_O Mundo Que Queremos)
- Quadrangle Institute
- Comida do Amanhã Institute
- State University of Amazonas
- Engineers Without Borders Brazil
- Federal Institute of Education, Science and Technology of Amazonas
- Oswaldo Cruz Foundation
- Universidade Federal do Tocantins / Federal university of Tocantins State

- Tabatinga Higher Studies Center of the State University of Amazonas
- Support Center for the Riverside Population of the Amazon
- Maranhao State University
- Western Pará Federal University
- Federal University of Espírito Santo
- Politize! - Civic Education Institute
- Fundação Getúlio Vargas / Getúlio Vargas Foundation
- Instituto Centro de Vida (ICV) / Centro de Vida Institute
- Imaflora
- Sociedade Rural Brasileira (SRB) / Brazilian Rural Society (SRB)
- Coordenação das Organizações Indígenas da Amazônia Brasileira – COIAB/COICA / Coordination of Indigenous Organizations of the Brazilian Amazon
- Embrapa
- Universidad Federal de São João del-Rei / Federal University of São João del-Rei
- CIRAD
- Empresa Brasileira de Pesquisa Agropecuária (EMBRAPA) / Brazilian Agricultural Research Corporation
- Brazilian Association of Sanitation & Special Waste Companies (ABRELPE)
- Federal University of Santa Catarina (Brazil)
- Unicamp
- Uakari Floating Lodge at Mamiraua
- Federal University of Pernambuco (Ufpe)
- Ecotropica Brazil
- Golden Lion Tamarin Association
- Roteiros de Charme Hotel Association
- Pontifícia Universidade Católica De Campinas / Pontifical Catholic University of Campinas
- Faculdade de Tecnologia do Estado de São Paulo / Faculty of Technology of the State of São Paulo
- Braztoa Brazilian Tour Operators Association
- University Anhemni Morumbi
- Universidade Estadual De Ponta Grossa / State University of Ponta Grossa
- University Dinamica Das Cataratas (UDC)
- Agriculture Research Institute, Brazil
- Alianza Cooperativa Internacional para las Américas / International Cooperative Alliance for the Americas
- Centro de Ciências Agrárias da Universidade Federal de Alagoas / Center of Agricultural Sciences of the Federal University of Alagoas
- Embrapa Cassava & Fruits
- Fundação Getúlio Vargas – Gvces / Getúlio Vargas – Gvces Foundation
- FA.VELA
- ChildFund Brasil
- Foundation for the Conservation of Biodiversity

Table S3: Chile-based TGI members

**Public Organizations**<br>(Entities governed by public law, which are located at the central, state, and local level, and national representations of international organizations)<br>• Ciudad de Santiago / Santiago City<br>• Ministerio de Agricultura Chile / Ministry of Agriculture Chile<br>• Ministerio de Medio Ambiente de Chile / Ministry of the Environment of Chile<br>• Government of Chile<br>• Instituto Nacional de Propiedad Industrial de Chile (INAPI) / National Institute of Industrial Property of Chile<br>• Ministerio de Vivienda y Urbanismo de la Republica de Chile / Republic of Chile Ministry of Housing and Urban Planning<br>• Ministerio de Medio Ambiente de Chile / Chile Ministry of Environment<br>• MAPS Chile
**Business Organizations**<br>(Entities governed by private law and which engage in for-profit activities)<br>• AVLA<br>• LATAM Airlines Group<br>• Viña Concha y Toro / Vineyard Concha y Toro<br>• MGC<br>• Sistemas Sustentables / Sustainable Systems<br>• ZEROCO2<br>• METRO DE SANTIAGO S.A. / Santiago Subway<br>• Redbus Urbano SA - Transdev Chile SA<br>• Tecnocom Chile S.A.<br>• Tecnocom Procesadora De Chile, S.A<br>• Cambio Climático y Desarollo (CC&D) / Climate Change and Development<br>• Deuman<br>• Numark Associates, Inc.<br>• POCH Ambiental S.A<br>• Solid Waste Group<br>• Premios FEDETUR / FEDETUR Awards<br>• Greenpartner<br>• Agentur Chile Travel Art<br>• Carnes Manada / Manada Meats<br>• Eficagua<br>• Huerto Puro<br>• Viñedos Orgánicos Emiliana / Emiliana Organic Vineyards<br>• Investigaciones y Datos SpA / Research and Data SpA<br>• MGC (Business)<br>• Sustainable Systems (Business)<br>• For the planet (Business)
**Civil Society Organizations**<br>(Entities governed by private law and which engage in non-profit activities as defined by Salamon and Anheier 1996)<br>• Adapt Chile

- Centro de Análisis en Políticas Públicas, Instituto de Asuntos Públicos, Universidad de Chile / Centre on Public Policy Analysis, Institute on Public Affairs, University of Chile
- Pontificia Universidad Católica de Chile (CLAPES UC) / Pontifical Catholic University of Chile (CLAPES UC)
- Observa Biobio Foundation
- Universidad del Desarrollo / Centro de Investigación en Sustentabilidad y Gestión Estratégica de Recursos / Facultad de Ingeniería / Development Institute / Center for Research in Sustainability and Strategic Resource Management / Faculty of Engineering
- Universidad Autónoma de Chile / Autonomous University of Chile
- Facultad de Ciencias Agrarias, Universidad Austral de Chile / Faculty of Agricultural Sciences, Southern University of Chile
- Pontificia Universidad Católica de Chile (UC) / Pontifical Catholic University of Chile (UC)
- Universidad Andrés Bello, Escuela de Ecoturismo/ University Andres Bello, School of Ecotourism
- Instituto de Investigaciones Agropecuarias / Institute of Agricultural Research
- Sociedad Chilena de la Ciencia del Suelo / Chilean Society of Soil Science
- Universidad de la Frontera
- Chile GBC Chile Green Building Council
- Corporacion Chilena del Documental / Chilean Documentary Corporation
- Cine Chile / Cinema Chile
- Adapt Chile (NGO)

Table S4: Colombia-based TGI members

Public Organizations
(Entities governed by public law, which are located at the central, state, and local level, and national representations of international organizations)

- Caquetá
- Guainía
- Guaviare
- Nariño
- Presidencia de Colombia / Presidency of Colombia
- Gobierno de Colombia / Government of Colombia
- CUÍTIVA
- TOTA
- Aquitania
- Ciudad de Medellín, Municipio de Medellín, Colombia / City of Medellín Municipality of Medellín, Colombia

Business Organizations
(Entities Governed By Private Law And Which Engage In For-Profit Activities)

- Banco Popular – Grupo Aval
- Bancolombia Sa
- Bancompartir Sa
- Seguros Bolivar / Insurance Bolivar
- Suramericana Sa
- Portafolio Verde
- Racafe & Cia S.C.A
- Siemens Sa
- Tecnocom Colombia S.A.S.
- Volvo Group Colombia
- Colombiana De Minerales / Colombian Minerals
- Corporación Cdt De Gas / Cdt Gas Corporation
- Ecotieda
- Promigas S.A. Esp
- Wes Tech Inc.
- Fedec National Meeting For Nature Based Tourism
- Federación Colombiana De Ecoparques / Colombian Federation Of Ecoparks
- Gelatam Sas
- Consejo Colombiano De Construcción Sostenible / Colombian Council For Sustainable Construction
- Semana Sostenible / Sustainable Week

Civil Society Organizations
(Entities governed by private law and which engage in non-profit activities as defined by Salamon and Anheier 1996)

- Ecotierra
- Fundacion Natura Colombia / Natura Foundation Colombia
- ECOTIERRA
- Fundacion Gaia Amazonas, Colombia / Gaia Amazonas Foundation, Colombia
- Organización Nacional de los Pueblos Indígenas de la Amazonia Colombiana – OPIAC / National Organization of the Indigenous Peoples of the Colombian Amazon

- Centro de Pensamiento Estratégico Internacional (Cepei) / Center for International Strategic Thinking
- Klimaforum Latinoamérica Network
- Universidad de Los Andes / University of Los Andes
- Centro de Estudios para el Desarrollo Sostenible Colombia (CEID Colombia) / Center of Studies for Sustainable Development Colombia
- Universidad de Cartagena / University of Cartagena
- Centro de Investigación en Agricultura Sostenible / Centre for Research on Sustainable Agriculture - CIPAV
- Universidad Atlantico / Atlantico University
- Asociación Colombiana de Desarrollo Sostenible y Ecología Industrial (ADSEI) / / Colombian Association for Sustainable Development and Industrial Ecology
- Universidad de Nariño / University of Narino
- Fundación País21/ Pais21 Foundation
- Universidad EAFIT / EAFIT University
- Instituto de Investigaciones de la Amazonia de Colombia / Amazon Research Institute of Colombia
- Academia Colombiana de Ciencias Exactas, Físicas y Naturales / Colombian Academy of Exact, Physical and Natural Sciences
- Universidad de Magdalena / University of Magdalena
- Instituto para el Desarrollo Sostenible de la Universidad del Norte / Institute for Sustainable Development at Universidad del Norte
- Instituto de Estudios Interdisciplinarios y Acción Estratégica para el Desarrollo / Institute of Interdisciplinary Studies and Strategic Action for Development - IdEAD
- Fundesabanas Foundation
- Universidad Tecnologica del Choco Diego Luis Córdoba / Technological University of Choco Diego Luis Córdoba
- University Foundation of Health Sciences / Fundación Universitaria de Ciencias de la Salud
- Asociación Colombiana de Educación al Consumidor / Colombian Association of Consumer Education
- Unipanamericana
- Universidad Industrial de Santander / Industrial University of Santander
- Federación Nacional de Cafeteros de Colombia (FNC) / National Federation of Colombian Coffee Growers
- Pacto Caquetá / Cauetá Covenant
- CIPAV
- Corporación Colombiana de Investigación Agropecuaria (CORPOICA) / Colombian Corporation for Agricultural Research
- Centro Internacional de Agricultura Tropical / International Center for Tropical Agriculture (CIAT)
- Federación Nacional de Cafeteros de Colombia (FEDERECAFE) / National Federation of Colombian Coffee Growers
- Federación Nacional de Cultivadores de Cereales (FENALCE) / National Federation of Cereal Growers
- Federación Nacional de Cultivadores de Palma de Aceite (FEDEPALMA) / National Federation of Oil Palm Growers
- Fundación para la investigación y desarrollo agrícola (Fidar) / Foundation For Agricultural Research And Development

- Universidad EAN / University EAN
- Universidad tecnológica del Chocó / Technological University Of Choco
- Centro Nacional de Produccion Mas Limpia Colombia (NCPC) / National Center for Cleaner Production Colombia
- Visit Sugamuxi
- Fundación Montecito / Montecito Foundation
- Fundación Humedales / Humedales Foundation
- Universidad Externado de Colombia / Externado University Of Colombia
- Fundación Natura Colombia / Natura Foundation Colombia
- Corporacion Biocomercio Sostenible / Sustainable BioCommerce Corporation
- Unidad para el Desarrollo de a Ciencia, la Innovación y la Investigación, Facultad de Derecho, Universidad Antonio Nariño, Duitama, Boyacá / Unit for the Development of Science, Innovation and Research, Faculty of Law, Antonio Nariño University, Duitama, Boyacá
- Centro de Estudios Regionales Cafeteros y Empresariales (CRECE) / Center for Regional Coffee and Business Studies
- ECOTIERRA

Table S5: Mexico-based TGI members

**Public Organizations**<br>(Entities governed by public law, which are located at the central, state, and local level, and national representations of international organizations)<br>• Aguascalientes<br>• Baja California<br>• Baja California Sur<br>• Campeche<br>• Chiapas<br>• Colima<br>• Guanajuato<br>• Hidalgo<br>• Jalisco<br>• Ciudad de Mexico / Mexico City<br>• Estado de Mexico / Mexico State<br>• Michoacán<br>• Nuevo León<br>• Oaxaca<br>• Querétaro<br>• Quintana Roo<br>• Sonora<br>• Tabasco<br>• Yucatán<br>• Gobierno de México Government of Mexico<br>• Secretaría de Relaciones Exteriores / Secretary of Foreign Relations<br>• Gobierno del Estado de Quintana Roo / Government of the State of Quintana Roo<br>• Gobierno del Estado de Yucatán / Government of the State of Yucatan<br>• Gobierno del Estado de Campeche / Government of the State of Campeche<br>• Gobierno del Estado de Tabasco / Government of the State of Tabasco<br>• Gobierno del Estado de Chiapas / Government of the State of Chiapas<br>• Secretaría de Salud de Michoacán / Secretary of Health of Michoacán<br>• Instituto de Derechos Humanos Francisco Tenamaxtli de la Comisión Estatal de Derechos Humanos Jalisco / Francisco Tenamaxtli Human Rights Institute of the Jalisco State Human Rights Commission<br>• Sistema de Transporte Colectivo / Collective Transportation System<br>• Fideicomisos Instituidos en Relación con la Agricultura (FIRA) / Trusts Established in Relation to Agriculture<br>• Secretaría de Agricultura Ganadería Desarrollo Rural Pesca y Alimentación (SAGARPA) / Secretary of Agriculture, Livestock, Rural Development, Fisheries and Food<br>• Municipio de Valladolid / Municipality of Valladolid<br>• Comisión Económica para América Latina y el Caribe (ECLAC) / Economic Commission for Latin America and the Caribbean<br>• SEMARNAT- Secretaría de Medio Ambiente y Recursos Naturales / Ministry of Environment and Natural Resources<br>• Programa Nacional De Auditoría Ambiental (PNAA), Mexico / National Environmental Audit Program, Mexico<br>• Municipio de San Miguel de Allende Gto

• SAGARPA<br>• Secretaría de Medio Ambiente y Recursos Naturales de los Estados Unidos Mexicanos / United Mexican States Secretariat of Environment and Natural Resources<br>• Comision National de Vivienda de Mexico (CONAVI) National Housing Commission (Conavi) of Mexico<br>• Secretaría de Desarrollo Agrario, Territorial y Urbano, SEDATU / Secretary of Agrarian, Territorial and Urban Development<br>• Sociedad Hipotecaria Federal de MéxicoFederal Mortgage Society of Mexico<br>• Secretaria de Ecologia y Gestion Ambiental / Secretary of Ecology and Environmental Management<br>• Comisión Nacional de Eficiencia Energética / National Commission for Energy Efficiency<br>• Instituto Nacional de Ecología y Cambio Climático de México (INECC) / Mexico National Institute of Ecology and Climate Change
**Business Organizations**<br>(Entities governed by private law and which engage in for-profit activities)<br>• Grupo Bimbo<br>• Bioconstructión y energía alternative / Bioconstruction and alternative energy<br>• Mabe<br>• Banco del Bajío, S.A. Institución de Banca Múltiple<br>• Banco Mercantil del Norte, S.A. Institución de Banca Multiple Grupo Financiero Banorte.<br>• Banco Regional S.A. (Banregio)<br>• BBVA México<br>• CIBanco S.A.<br>• Compartamos Banco<br>• Grupo Financiero Banamex<br>• Monex Grupo Financiero<br>• Quálitas Compañía de Seguros<br>• Unifin Financiera, S.A.B. de C.V.<br>• Cemex<br>• Grupo Financiero Banorte<br>• Mexico2<br>• COYSEP Consultoría y Servicios Especializados SA de CV. / COYSEP Consulting and Specialized Services<br>• THREE Consultoría Medioambiental / THREE Environmental Consulting<br>• SOLBEN<br>• CEMEX<br>• Cámara de Comercio Internacional México (ICC México) / International Chamber of Commerce México<br>• Grupo Ado - Mobility Ado<br>• Scania De Mexico S.A. DE C.V.<br>• TECNOCOM, Telefonía y Redes De México, S.A. de C.V. / TECNOCOM Telephone and Networks of Mexico<br>• Fundacion Produce Michoacan A.C. / Produce Michoacan Foundation<br>• AARSP<br>• Agrobal Servicios / Agrobal Services<br>• AgroBIO

• Consejo Empresarial de la Industria del Maíz y sus Derivados / Business Council of the Corn Industry and its Derivatives<br>• Grupo Agro-empresarial Cresa / Cresa Agro-business group<br>• Makala<br>• Monsanto<br>• NOVASEM<br>• Semillas Moreno Retis / Moreno Retis Seeds<br>• Plataforma Mexicana de Carbono Mexican (MexiCO2) / Carbon Platform<br>• AlterLat Energia Alternativa de Latinoamerica / Alternative Energy of Latin America<br>• Biogas de Juarez / Juarez Biogas<br>• Cappy and Associates, Mex. SA de CV<br>• Enersus Mexico<br>• Genegas S.A. De C.V.<br>• Green Group Mexico<br>• Grupo Empresarial de Energía y Medio Ambiente / Energy and Environment Business Group<br>• Mor Hydro<br>• PRUHESA<br>• Tecnologia Ambiental de Mexico SA de CV / Environmental Technology of Mexico SA de CV<br>• URMO Ingeniería Integral, SA<br>• Journey Mexico<br>• Totonal Viajes Que Iluminan<br>• Mesones Sacristia Puebla<br>• Holiday Inn Mexico Coyoacan<br>• Hotel Ocean Coral y Turquesa<br>• Ambient<br>• Biofabrica Siglo XX SA DE CV<br>• Fomex<br>• Fideicomiso para el Ahorro de Energía Eléctrica (FIDE) / Trust Fund for Electric Energy Savings<br>• THREE Consultoria Ambiental / THREE Environmental Consulting
**Civil Society Organizations**<br>(Entities governed by private law and which engage in non-profit activities as defined by Salamon and Anheier 1996)<br>• El Buen Socio<br>• Universidad Autonoma Chapingo / Chapingo Autonomous University<br>• Instituto Nacional de Ecología y Cambio Climático (INECC) / National Institute of Ecology and Climate Change<br>• Grupo de Financiamiento Climático para América Latina y el Caribe / Climate Finance Group for Latin America and the Caribbean<br>• ITDP México<br>• Asamblea Mixe para el Desarrollo Sostenible A.C. / Mixe Assembly for Sustainable Development<br>• Grupo Ecológico Sierra Gorda IAP / Sierra Gorda Ecological Group<br>• Pronatura Sur, A.C.<br>• Reforestamos México A.C.

- Tecnológico de Monterrey
- Sierra Gorda Ecological Group
- Universidad de Guadalajara, Instituto de Investigación en Políticas Públicas y Gobierno / University of Guadalajara, Institute for Research in Public Policy and Government
- Instituto Global para la Sostenibilidad-EGADE Business School at Tecnologico de Monterrey / Global Institute for Sustainability EGADE Business School at Tecnologico de Monterrey
- Universidad Politécnica de Altamira / Polytechnic University of Altamira
- Universidad Anahuac / Anahuac University
- Universidad Popular de Chontalpa / Popular University of Chontalpa
- Laboratorio Nacional de Políticas Públicas / National Public Policy Laboratory
- Universidad Autonoma de Nuevo Leon / Autonomous University of Nuevo Leon
- Universidad CIE / CIE University
- Network of Sustainability Energy, Environmental and Social / Red de Sostenibilidad Energética, Ambiental y Social
- Instituto Mexicano de Ciudades Inteligentes Sostenibles / Mexican Institute of Sustainable Smart Cities
- Universidad Juárez Autónoma de Tabasco / Juárez Autonomous University of Tabasco
- Universidad Autónoma del Estado de México / Autonomous Mexico State University
- Red de contaminación atmosférica y cambio climático / Atmospheric Pollution and Climate Change Network
- Universidad Tecmilenio / Tecmilenia University
- Universidad de Sonora / University of Sonora
- Universidad Iberoamericana / Ibero-American University A.C.
- Facultad de Ingeniería Mecánica y Ciencias Navales, Universidad Veracruzana / Faculty of Mechanical Engineering and Naval Sciences, Veracruzana University
- Universidad Nacional Autónoma de México (UNAM) / National Autonomous University of Mexico
- University of Guadalajara / University of Guadalajara
- Universidad Tecnológica de Tabasco / Technological University of Tabasco
- Universidad Tecnológica de Altamira / Technological University of Altamira
- Campus Guanajuato de la Universidad de Guanajuato / Guanajuato Campus of the University of Guanajuato
- Instituto Internacional de Recursos Renovables / International Renewable Resources Institute
- Centro de Investigación Científica y Educación Superior en Ensenada, Baja California, México / Center for Scientific Research and Higher Education at Ensenada, Baja California, México
- Universidad Autonoma de Queretaro / Autonomous University of Querétaro
- Oxfam Mexico
- Universidad Autonoma de Coahuila / Autonomous University of Coahuila
- Universidad Autónoma de Baja California / Autonomous University of Baja California
- Instituto Mora / Mora Institute
- Instituto Tecnológico Superior Centla / Centla Higher Technological Institute
- Universidad Autónoma de Yucatán / Autonomous University of Yucatan

- Universidad de Celaya / University Of Celaya
- Consorcio para la Investigación, Innovación y Desarrollo de las Tierras Áridas / Consortium for Research, Innovation and Development of the Drylands
- Universidad Tecnológica del Área Metropolitana del Valle de México / Technologic University of the Metropolitan area of the Valley of Mexico
- Higher Technological Institute of Teposcolula/ Instituto Tecnológico Superior de Teposcolula
- Universidad Politécnica de Yucatán / Polytechnic University of Yucatan
- Universidad Autonoma Metropolitana / Autonomous Metropolitan University
- Autonomous University of Baja California Sur / Autonomous University of Baja California Sur
- ECOYDES A.C.
- Universidad Iberoamericana Puebla / Iberoamerican University Puebla
- Instituto de Educación Digital del Estado de Puebla / Institute of Digital Education of the State of Puebla
- Universidad Autonoma Metropolitana / Autonomous Metropolitan University
- Centro de Investigación y Enseñanza de la Economía / Center for Research and Teaching of Economics
- Universidad Autonoma de la Ciudad de Juarez / Autonomous University of Ciudad Juarez
- Universidad La Salle / La Salle University
- Universidad Autonoma De San Luis Potosi / Autonomous University of San Luis Potosí
- Colegio de Estudios Científicos y Tecnológicos del Estado de Hidalgo CECyTE Hidalgo / Hidalgo State College of Scientific and Technological Studies
- Instituto Tecnológico Superior de Huichapan Superior / Technological Institute Of Huichapan
- Universidad María Esther Zuno de Echeverría / University María Esther Zuno de Echeverría
- Benemérita Universidad Autónoma de Puebla / Meritorious Autonomous University of Puebla
- Universidad Tecnológica Metropolitana / Metropolitan Technological University
- Centro de Investigación y de Estudios Avanzados del Instituto Politécnico Nacional / Center for Research and Advanced Studies of the National Polytechnic Institute
- Fundacion Pensar / Pensar Foundation
- ITESO, Universidad Jesuita de Guadalajara / Jesuit University of Guadalajara
- Coordinación Universitaria de Observatorios de la Universidad Veracruzana / University Coordination of Observatories of the Universidad Veracruzana
- Universidad Tecnológica de los Valles Centrales de Oaxaca / Technological University of the Central Valleys of Oaxaca
- Universidad Anáhuac Mayab / Anáhuac Mayab University
- UNLA
- Universidad Riviera / Rivera University
- Universidad Incarnate Word, Campus Bajío / Incarnate Word University Campus Bajío
- Universidad Autónoma del Carmen / Autonomous University of Carmen
- Instituto Universitario de Yucatán Sc / University Institute of Yucatan Sc
- Universidad Cuauhtémoc Puebla / Cuauhtémoc Puebla University

- Instituto Tecnológico Superior de Zacapoaxtla / Higher Technological Institute of Zacapoaxtla
- Universidad Anáhuac Querétaro / Anáhuac Querétaro University
- Universidad Carolina / Carolina University
- Unidad para la Inclusión Educativa y Atención a la Diversidad de la Universidad Autónoma del Estado de Morelos / Unit for Educational Inclusion and Attention to Diversity of the Autonomous University of the State of Morelos
- Facultad de Ingeniería de la Universidad Autónoma de Campeche / Faculty of Engineering of the Autonomous University of Campeche
- Universidad Quetzalcóatl en Irapuato / Quetzalcoatl University in Irapuato
- Universidad Veracruzana / Veracruzana University
- Universidad La Salle Pachuca / La Salle Pachuca University
- Centro de Investigación de la Mujer en la Alta Dirección / Center for Research on Women in Senior Management
- CECyT 16 del Instituto Politécnico Nacional Campus Hidalgo (Unidad de Tecnología Educativa y Campus Virtual) / CECyT 16 of the Hidalgo Campus National Polytechnic Institute (Educational Technology Unit and Virtual Campus)
- Universidad Politécnica de Santa Rosa Jáuregui / Polytechnic University of Santa Rosa Jáuregui
- Universidad Autónoma de Tamaulipas / Autonomous University of Tamaulipas
- CEMDA
- Impacto Café
- El Colegio de la Frontera Sur / Frontera del Sur School
- Centro de Investigación y de Estudios Avanzados del Instituto Politécnico Nacional (Cinvestav) / Center for Research and Advanced Studies of the National Polytechnic Institute (Cinvestav)
- Instituto Nacional de Investigaciones Forestales Agrícolas y Pecuarias (INIFAP) / National Institute of Agricultural and Livestock Forestry Research
- Universidad Autónoma Metropolitana (UAM) / Metropolitan Autonomous University
- Universidad Juárez del Estado de Durango/ Juárez University of the State of Durango
- Universidad de San Carlos / University of San Carlos
- Universidad de Guadalajara / University of Guadalajara
- Laboratorio Nacional de Genómica para la Bioversidad (LANGEBIO) / National Laboratory of Genomics for Bioversity (LANGEBIO)
- Universidad Nacional Autónoma de México / National Autonomous University of Mexico
- Tecnológico de Monterrey
- Universidad Autónoma Agraria Antonio Narro / Autonomous Agrarian University Antonio Narro
- Colegio de Postgraduados / Postgraduate College
- Universidad Autónoma Chapingo / Chapingo Autonomous University
- Universidad Autónoma Agraria / Autonomous Agrarian University
- Instituto Tecnológico de Tuxtla Gutiérrez (ITTG) / Technological Institute of Tuxtla Gutiérrez (ITTG)
- Fondo para la Paz / Fund for Peace

- Asociación para la Agricultura Sostenible en Base a Siembra Directa A.C. (ASOSID) / Association for Sustainable Agriculture based on Direct Sowing A.C. (ASOSID)
- Sistema Producto Maíz del D.F. / D.F. Corn Product System.
- Instituto Internacional de Recursos Renovables - México (IRRI) / International Renewable Resources Institute - Mexico (IRRI)
- Universidad Michoacana de San Nicolás de Hidalgo /Michoacan University of San Nicolas de Hidalgo
- Universidad Autónoma De Ciudad Juárez / Autonomous University of Ciudad Juarez
- Glocal Tourism Consulting
- Instituto de Estudios Superiores de Tamaulipas / Institute of Higher Eduaction of Tamaulipas
- Sociedad Amigos del Lago de Chapala A. C. / Amigos del Lago de Chapala Society
- Universidad Tecmilenio / Tecmilenio University
- Rec_ Recursos Sustentables / Rec_ Sustainable Resources
- Fundación Helvex / Helvex Foundation
- Mexico Tourism Board
- Universidad Anáhuac del Sur / Anahuac del Sur University
- Universidad Intercontinental / Intercontinental University
- Pronatura Sur, A.C.
- Universidad de Quintana Roo / Quintana Roo University
- Instituto Nacional de Investigaciones Forestales, Agrícolas y Pecuarias / National Institute of Forestry, Agriculture and Livestock Research
- Vía Orgánica
- Asociacion Nacional de Agricultura de Conservación (ANAC) / National Conservation Agriculture Association (ANAC)
- Centro International de Mejoramiento de Maíz y Trigo (CIMMYT) / International Maize and Wheat Improvement Center (CIMMYT)
- Estampa Verde
- ALENER Alianza para la Eficiencia Energética / Alliance for Energy Efficiency
- ITSPM Instituto Technologico Superior P’urhépecha Mexico / Higher Technological Institute P'urhépecha Mexico
- Centro Regional del Convenio de Basilea para Centroamerica y Mexico / Regional Center of the Basel Convention for Central America and Mexico
- Cinema Planeta
- Festival Internacional de Cine en Guadalajara / International Film Festival in Guadalajara
- WRI México
- Grupo de Financiamiento Climático para América Latina y el Caribe / Climate Finance Group for Latin America and the Caribbean (NGO)
- ITDP México (NGO)
- Universidad de Monterey / University of Monterrey
- CGIAR Excellence in Breeding (EiB)

Table S6: Peru-based TGI members

**Public Organizations**<br>(Entities governed by public law, which are located at the central, state, and local level, and national representations of international organizations)<br>• Amazonas<br>• Huánuco<br>• Loreto<br>• Madre de Dios<br>• Piura<br>• San Martín<br>• Ucayali<br>• Gobierno del Perú / Government of Peru<br>• Amazonas, Peru<br>• Huanuco, Peru<br>• Loreto, Peru<br>• San Martin, Peru<br>• Ucayali, Peru<br>• Madre de Dios, Peru<br>• Instituto Nacional de Innovación Agraria (INIA) / National Institute of Innovation<br>• Ministerio de Agricultura / Ministry of Agriculture<br>• Ministerio de Vivienda, Construcción y Saneamiento de la República del Perú / Republic of Peru Ministry of Housing, Construction and Sanitation<br>• Swisscontact Peru Fundación Suiza para la Cooperación Técnica / Swisscontact Peru Swiss Foundation for Technical Cooperation<br>• CIAM - Consejo Interregional Amazonico / Amazon Interregional Council
**Business Organizations**<br>(Include entities governed by private law and which engage in for-profit activities)<br>• Pronatur S.A.C.
**Civil Society Organizations**<br>(Include entities governed by private law and which engage in non-profit activities as defined by Salamon and Anheier 1996)<br>• Chirapaq- Centro de Culturas Indígenas del Perú / Center for Indigenous Cultures of Peru<br>• Association for Research and Integral Development – AIDER / Asociación para la Investigación y el Desarrollo Integral<br>• Universidad Señor de Sipan / Señor de Sipan University<br>• Instituto de Investigaciones de la Amazonía Peruana / Peruvian Amazon Research Institute<br>• Asociación Amazónicos por la Amazonía- AMPA / Amazonians for the Amazon Association<br>• Practical Action<br>• Universidad del Pacífico, Centro de Investigación / Pacific University, Research Center<br>• Movimiento Ciudadano Contra el Cambio Climático (MOCICC) / Citizen Movement Against Climate Change (MOCICC)<br>• Colegio Profesional de Sociólogos del Perú / Professional College of Sociologists of Peru<br>• Centrum PUCP

- Universidad de Lima / Lima University
- EcoSwell
- Derecho, Ambiente y Recursos Naturales (DAR) / Law, Environment and Natural Resources
- Centro Internacional de la Papa / International Potato Center
- Universidad Nacional Agraria (UNA) / National Agrarian University
- Asociación ANDES / ANDES Association
- Sociedad Peruana de Derecho Ambiental / Peruvian Society for Environmental Law
- Instituto de Estudios Peruanos / Institute of Peruvian Studies

S7: Logistic regression with Chilean MNCs as the reference category

Country Dummies	Odds Ratios	Standard Errors
Argentina	1.350	0.905
Brazil	0.984	0.475
Chile	Reference	
Colombia	2.045	1.250
Mexico	1.479	0.745
Peru	2.250	3.330
Observations	215	
Wald chi2(5)	3.23	

Notes: * $p < 0.10$, ** $p < 0.05$; *** $p < 0.01$.
